# The Effect of Adaptation on the Tuning Curves of Rat Auditory Cortex

**DOI:** 10.1371/journal.pone.0115621

**Published:** 2015-02-26

**Authors:** Mohsen Parto Dezfouli, Mohammad Reza Daliri

**Affiliations:** Biomedical Engineering Department and Iran Neural Technology Centre (INTC), Faculty of Electrical Engineering, Iran University of Science and Technology (IUST), Narmak, 16846–13114 Tehran, Iran; University of Southern California, UNITED STATES

## Abstract

Repeated stimulus causes a specific suppression of neuronal responses, which is so-called as Stimulus-Specific Adaptation (SSA). This effect can be recovered when the stimulus changes. In the auditory system SSA is a well-known phenomenon that appears at different levels of the mammalian auditory pathway. In this study, we explored the effects of adaptation to a particular stimulus on the auditory tuning curves of anesthetized rats. We used two sequences and compared the responses of each tone combination in these two conditions. First sequence consists of different pure tone combinations that were presented randomly. In the second one, the same stimuli of the first sequence were presented in the context of an adapted stimulus (adapter) that occupied 80% of sequence probability. The population results demonstrated that the adaptation factor decreased the frequency response area and made a change in the tuning curve to shift it unevenly toward the higher thresholds of tones. The local field potentials and multi-unit activity responses have indicated that the neural activities strength of the adapted frequency has been suppressed as well as with lower suppression in neighboring frequencies. This aforementioned reduction changed the characteristic frequency of the tuning curve.

## Introduction

Neural adaptation is a common phenomenon which has been extensively observed in the mammalian sensory area such as visual [1,2], auditory [3,4,5], somatosensory [6], and so forth. Commonly, adaptation tends to suppress the neuronal activities in sensory systems. Adapting to the environment frequent stimuli such as light, smell, and sound is a vital brain function that the lack of it can be very disturbing. This mechanism causes some variations in neural properties against reputation to decrease the attention to the frequent stimulus. Particularly it leads to increase the neural sensitivity against unexpected changes for deviance detection [3]. The recent stimulus history affects the activities of cortical neurons. In the auditory pathway, adaptation depends on various stimulus parameters like frequencies distance, intensity, Inter-Stimulus Interval (ISI) and probability occurrence of the stimulus. In addition, recent studies have indicated that in an audio sequence the randomness of stimuli presentation affects significantly the neural responses of them [7].

Research on adaptation and change detection goes back to evoked potential signals in mismatch negativity (MMN) studies, which indicated this ability in human [8], monkey [9] and cat [10], (for review see [11]). MMN is a component of Event Related Potential (ERP) that occurs in the response of rare stimulus in a sequence. In auditory, two frequencies with almost similar responses were used in an oddball paradigm. An oddball paradigm contains two frequencies, which are presented with different probability. In a sequence, one tone is presented repeatedly as standard stimulus and one other is presented rarely, and in other sequences, the role is exchanged. The difference between the frequency responses in standard and rare state results into the MMN wave. The MMN arises irrelevant to subject attention and has more clinical usage [8, 12]. In recent years, oddball paradigm has been extensively used in electrophysiological studies. The researchers have determined a phenomenon like MMN in the auditory cortical neurons, which is called Stimulus-Specific Adaptation (SSA) [3, 13, 14]. In early studies, it was assumed that this phenomenon was based on cortical processes; however in next year’s it was determined in other subcortical pathway neurons too, such as Inferior Colliculus (IC) [4,15] and Medial Geniculate Body (MGB) [16]. Ventral division of the thalamic nuclear MGB (MGv) has contributed in SSA. In SSA mechanism, this area is the major path into the auditory cortex [17]. The greatest amount of adaptation effect belongs to the auditory cortex neurons. SSA causes a significant declining in neural responses of frequent stimulus. It represents that a novel sound releases stronger response than the usual one.

Tuning curve is an important response curve, which expresses the relation between responses of sensory neurons to peripheral stimuli, and is assumed as a criterion for the evaluation of neurons function in different brain areas. In auditory system, tuning curve identifies the response of one or some neurons for sound stimuli at different frequencies-intensity combinations. The frequency, which creates responses in minimum intensity, is called Characteristic Frequency (CF). The studies declared different types of tuning curves (U shape, O shape, multi peak, V shape, and so on) in auditory cortical and subcortical regions [18].

Most previous studies of SSA in different auditory cortical and subcortical neurons have described the adaptation effects to repetitive sounds in an oddball design with two different probability occurrences. Recent research [19] reported that in a sequence, the neighboring frequencies of a specific sound stimulus influence the response of neurons. It showed that the neural responses of a specific stimulus with a constant probability varied in different sequences contained of diverse peripheral stimuli.

The objective of this study was to determine the relationship between responses of a sound stimulus in a control sequence and an adapted sequence. For this purpose, we considered SSA in a more complex pattern with a paradigm, which is consisted of pure tones with different frequency and intensity combinations. We designed a paradigm comprised of two audio sequences and used multiunit recording techniques to explore the effects of adaptation on the auditory tuning curves in rat primary auditory cortex. We found that adaptation shifts the auditory tuning curve toward higher-level intensities. We compared each frequency response in two sequences. The response to the adapter stimulus was decreased significantly. However, the response of the neurons to the neighboring frequencies was not decreased significantly. Hence, in a sequence, the adaptation to a specific stimulus around CF causes tuning curve to shifts toward the higher threshold and changes the CF.

## Materials and Methods

### Ethical approval

All experiments were carried out in Iran Neural Technology Center (INTC) at Iran University of Science and Technology (IUST) with the approval of, and using methods conforming to the standards of the ministry of health and medical education. The animals are maintained in the cages in facilities of the Neural Technology Center. The animal care and use committee of Neuroscience Research Laboratory, Iran University of Science & Technology approved all surgical procedures and experimental protocols in strict accordance with the recommendations in the Guide for the Care and Use of Laboratory Animals of the National Institutes of Health. All surgery was performed under urethane pentobarbital anesthesia, and all efforts were made to minimize suffering. At the end of experimental period, the animal was scarified under ether anesthesia.

### Animal preparation

The data were recorded from the left auditory cortex of 15 adult male and female Wistar rats weighing 250–350 gr. Anesthesia was performed using an intramuscular injection of urethane (1.5 g/Kg, 20% solution i.p) and supplementary doses (0.5 g/Kg, i.p.) as required. Urethane is a proper drug for anesthetized recording because it has less effect on the neural activities in comparison with other pentobarbital as well as stable longtime remaining anesthesia [20]. The hair directly over the surgical site was clipped. Then animal was placed in the stereotaxic instrument and his/her head was fixed with a head holder [21], that left the scalp in such a way that his ears were remained free. 1 ml lidoccain 2% was injected under the head skin. Before incision the skin, the surgical site was cleaned with alcohol. The skin and muscles of the left temporal part of the skull were removed and a craniotomy was performed over the location of primary auditory cortex (3–6.5mm posterior to and 6–7.5mm lateral to Bregma). We used the medical atlas of rat brain to expose the local area of the brain [22]. In the next step, saline was applied on the brain surface to protect it from drying. In addition, two screws were implanted on the skull as ground and reference points for recording. At the end of experimental period, the animal was scarified under ether anesthesia.

### Electrophysiological recording

A linear multi-electrode array, consisting of four tungsten electrodes (FHC, 5M, USA) with 200 um distance were used for extracellular recording. The electrodes were directed into the cortex using a Microdrive (SM-21, Narishige, Japan). The LFP and MUA recordings were performed using the USB-ME64-PGA recording system (Multichannel system; Germany). Furthermore, an on-line data visualization was controlled with the Multichannel software ‘MC-Rack’. The recorded raw signals of each channel (with 10 kHz sampling rate) were initially pre-amplified by an eight-Channel Miniature Preamplifier (MPA). Next, the amplified signals were filtered between 1–5000 Hz and then were amplified again with the gain of 1000 with Programmable Gain Amplifier (PGA) device. Finally, in order to display and record the data for off-line analyses, the signals were transmitted to the computer.

### Acoustic stimuli

All used stimuli were pure tones with duration of 50 ms, 5 ms rise-fall time (linear ramps) and 300 ms Inter-Stimulus Interval (ISI, onset to onset) that were generated using MATLAB. Generated audio events were stored in a memory card (MMC) and were played simultaneously with a trigger pulse that was send to the digital input of recording system in order to represent the onset of stimulus. Experiments were conducted in a shielding room. The pure tones were presented through an insert phone (ER3) that was placed into the rat’s right ear canal by a foam. This insert phone covers the human hearing frequencies band (20 Hz–20 kHz), so we used the same band of frequencies for this study.

### Experimental protocol

In order to detect the selective neurons a Broad Band Noise (BBN) bursts were presented with 300 ms duration (frequency between 1–20 kHz) and 500 ms ISI at amplitude of 50 dB. Considering all four electrodes, only the channels which their average LFP amplitude were large enough, have been recorded. Then, different frequency-level combinations of pure tones were presented (50 ms duration, 300 ms ISI). Various arrangements of 11 frequencies (between 200 Hz–20 kHz, with the frequency difference of f = (f2-f1)/f1 = 44%) $\Delta f=\frac{f2-f1}{f1}=44\%$) and 7 intensities (70–10 dB, 10dB steps) were considered to measure the Frequency Response Area (FRA) of every recording site. Each frequency-level combination was presented 10 times in a quasi-random sequence. Because of several combinations of intensities and frequencies, the sequences had to be as short as possible in order to get comparable data from two paradigms in the same recording site. Based on FRA of every single site which was found by the average LFP responses, five selected frequencies around the CF were considered for the main paradigm. For each site, the five selected frequencies in four above intensities (40–70 dB by 10dB steps) organized 20 frequency-level combinations. Theses pure tone combinations were presented and compared in the two sequences.

**Control sequence (C)**. In the first sequence, 20 selected combinations (five selected frequencies in four levels) were presented randomly. Each frequency-level combination was presented in 30 trials. LFP and MUA responses of all combinations were averaged across trials and then the tuning curve (control condition) was achieved.

**Fig 1:**
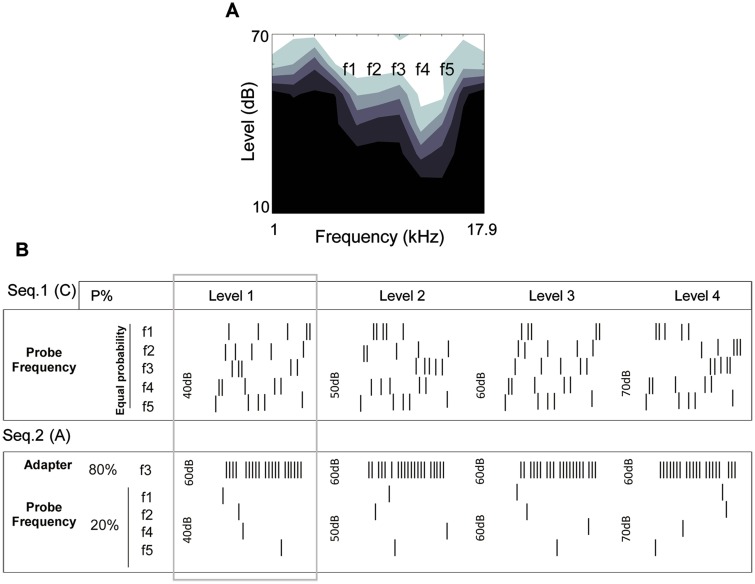
Adaptation paradigm. A, Tuning curve of one site and five proper frequencies that selected around the charactristic frequency. B, Two sequences that were utilized for investigating the adaptation effect. Seq.1 is control sequence that the selected frequencies were presented with equal probability in four intensities. Each frequency-level combination presented in 30 trials. Seq.2 is adapting sequence. In this prototype from selected frequencies, the middle one (f3) was chosen as an adapter frequency and the others were assumed neighboring frequencies. This adapter (middle frequency in level 60 dB) was presented randomly between the selected frequencies in four intensities, with the probability of 80% of all. The rectangular shows the basic of these two sequences in one specific intensity.

**Adapting sequence (A)**. In the second sequence, to find the adaptation effects on the tuning curve, the middle frequency (f3) was selected as the adapted frequency. This frequency at the level of 60 dB was considered as the adapter and the other combinations were assumed its neighborings. In this sequence, the adapter tone was presented four times per each combination. Audio tones were presented in a quasi-random sequence. Therefore, the adapted stimulus occupied 80% of all tone probability in this sequence. By considering this sequence, the adapting condition tuning curve can be achieved. Fig. 1 illustrates a schematic of the main paradigm which is consisting of these two sequences (seq.1 and seq.2).

The pure tones were presented in 30 trials and in a random sequence. According to the recent experiments, 25 presentations of each stimulus were enough to estimate an average response with a reasonable signal to noise ratio [19]. In order to ensure the stability of recording conditions, first sequence was presented once before the adapted sequence and once after it. The sites with more than 30% variation in responses were excluded. Since, different parameters of a sound can affect the neural responses of it [23,24], to find the adaptation effect, the duration and ISI of stimuli were assumed constant in the two sequences.

### Data Analysis

LFP and action potentials were recorded from four channels simultaneously. The data were analyzed using Matlab. For LFP, the raw data was passed from a low pass filter with cutoff frequency of 300Hz. Then all responses were aligned on the stimulus onset, and their baselines were corrected. In order to modify the baseline as close as possible to the response onset, the average response of the first 5 ms duration from the stimulus onset was subtracted from it. The first latencies were always longer than 8 ms, [19]. Maximum variation of voltage through average was considered during onset to 80 ms after onset to quantify the response strength. Because of a delay tendency in the adapted response, an extra timing duration for analysis related to stimulus duration was considered (80 ms compared to 50 ms). The standard error of the mean (s.e.m.) was considered as a criterion for measuring variability of the response. For MUA analyses, the raw signals were filtered using band pass filter with cut-off frequencies of 300 and 3000 Hz.

For automatic spike-detection from raw signals, a threshold should be set. This can be set, as a multiple of the standard deviation of the signal [25]. In this study, according to the recorded signals twice of the standard deviation was considered as a threshold for spike detection.

The resulted spike trains were aligned regarding the stimulus onset, smoothed with a 10 ms Hamming window, and averaged.

In order to quantify the adaptation effects, the percentage of difference between response activities of control and adapting conditions was measured. The adaptation changes were characterized by a criterion that was called Adaptation Index (AI) in an analysis similar to SSA index (SI) that was employed in [3, 13]. This measurement was calculated based on LFP variations and spike counts. It was defined as the following formula:

$$AI=\frac{C(fi)-A(fi)}{C(fi)+A(fi)}$$ (1)

where the parameters C(f_i_) and A(f_i_) represent the response strength of a frequency f_i_ in control and adapting sequences (seq.1, seq.2) respectively. The AI was used for both LFP and MUA comparison. It measures the difference of each frequency response in control and adapted sequences. For LFP analyses, the difference between maximum and minimum of LFP response during 0–80 ms after onset was measured. It identified the response strength of the neurons faced to each stimulus. In terms of firing rate, the response strength of each frequency f_i_ was considered as the firing rate value of first 80 ms from the onset. Therefore, the AI indicates how neural responses are changed in the desired two sequences.

The FRA of each site was separately mapped for two sequences. In order to do better comparison of the variation of the tuning curves in two sequences, the border of tuning curves were achieved by defining a threshold. This threshold is the minimum response of each frequency and declares the limited area of responses. This border usually is defined as mean spikes-per-presentation ± 1 SD [18], [15]. The threshold criterion for determining the tuning curve borders was defined as following:

T=M(y)+SD(y)+0.1×Max(y) (2)

where ‘y’ is the neural activities related to firing rate in MUA or voltage variation in LFP responses. ‘M’ is the mean value of neural responses and ‘SD’ is the standard deviation of the neural responses. Therefore the proper threshold (T) was characterized and the neural activities more than it were determined to specify the tuning curve.

## Results

Extracellular recordings from primary auditory cortex of urethane-anesthetized rats show that adaptation to specific stimulus near preferred or CF causes a reduction in neural response and dwindle FRA. The results demonstrate that the suppression of neural activities was specifically significant in the response of the adapter frequency.

All data were recorded from primary auditory cortex (A1) of 15 rats (n = 167 recording sites). Local Field Potential (LFP) and Multi-unit Activity (MUA) were simultaneously collected. Similar to the other sensory systems, the responses of auditory cortex neurons were reduced due to repetitive stimulus. In the current study, we considered this repetition effect on the auditory tuning curves. Because of some system constraints, we considered only neurons in A1 which response to low frequency tones. The neurons with CF between 200 Hz—20 KHz were selected for recording.

### Adaptation affects the LFP variation and MUA histogram

**Fig 2:**
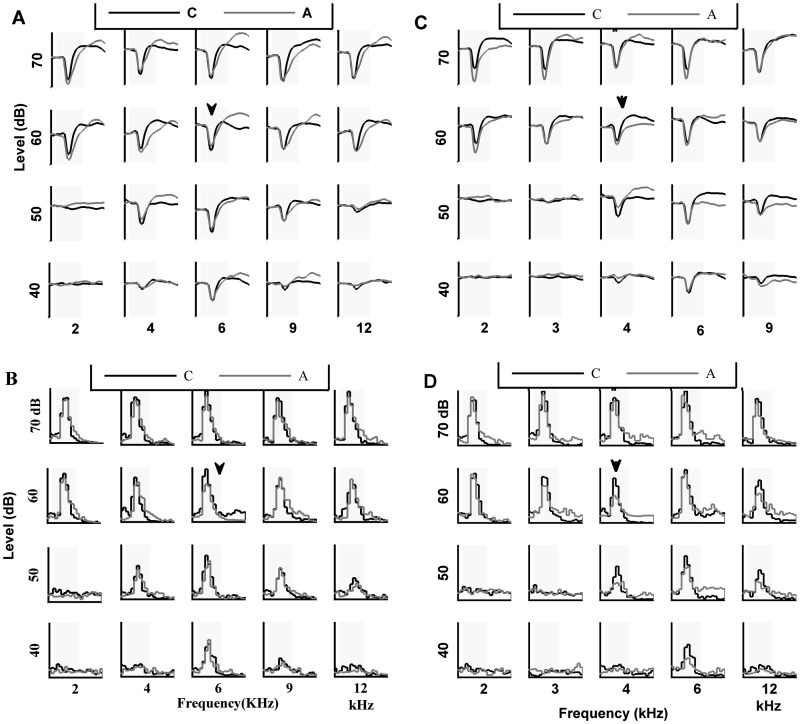
Comparison of local field potential (LFP) and multiunit activities (MUA) responses of control and adapted sequences in a single site. A. LFP responses of the selected combinations (five selected frequency in four intensities) in the control (black line) and adapted (gray line) sequences at a single recording site. B. MUA responses of theses two sequences (control; black line, adapted; gray line) that were identified with PSTH. C,D. Neural responses of the same site, similar to A,B with different adapter. In all figures, x axis is time duration between 10ms before to 90ms after stimulus onset, y axis presents the strength of the responses, the transparent window shows 50 ms stimulus presentation and the black triangular marks indicate the adapter stimulus.

For each recording site, the control and adapting sequences (illustrated in material and methods) were presented. The response of each frequency-amplitude combination was compared in the two conditions. The onset of responses was aligned and the responses were averaged across trials for time interval between-10 ms to 90 ms after the stimulus onset. Fig. 2A shows the responses of selected frequencies in a single site. It shows a reduction in the responses of stimulus in the adaptation sequence compared to the control one. In Fig. 2B, Pre-stimulus Time Histogram (PSTH) with 10 ms bin size declared MUA responses of these frequencies. Fig. 2C, 2D show the LFP and MUA responses of this recording site with another adapter.

### Effect of adaptation on the tuning curve in single site

**Fig 3:**
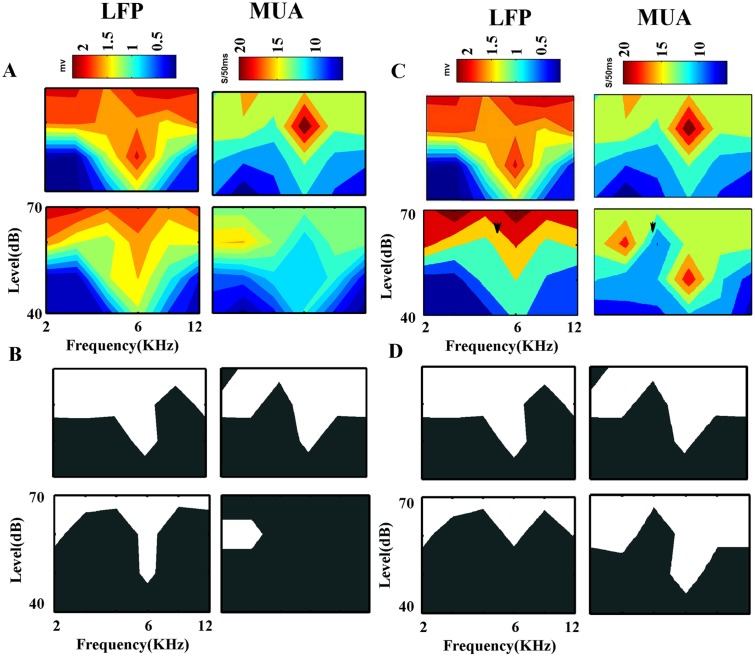
Comparison between the tuning curves of control and adapting conditions in one site. A. The First row shows FRA of one site that has been achived using LFP and MUA in the control sequence and the second row shows the adapting sequence responses. The adapter was marked by a V-shape marker. B. The tuning curves of this site in two sequences that were achieved by LFP and MUA responses and calculated according to a threshold that was defined. C,D. The FRAs and tuning curves of this site (similar to A,B) that used different adapter in its second sequence.

In order to compare the neural activities in the two aforementioned states, the tuning curves of neural activities have been considered. For each sequence (control and adapting sequences), analyses were performed based on the LFP and MUA and consequently two tuning curves were characterized. LFP voltage change and spike firing rate have been used in order to measure the FRA and the tuning curve. Considering LFP signals, voltage difference was calculated during 80 ms after stimulus onset in each frequency-level combination and it was assumed as the response strength. This quantity was averaged across all trials for each combination. Moreover, it mapped in a contour consisted of different frequency and level responses. The same calculation was performed by considering the firing rate of this duration (80 ms) in each combination. In Fig. 3A, the FRAs of two sequences have been compared. The first rows are FRA of control sequence and the second rows relate to the FRA of adapting sequence. For comparison of tuning curves, in Fig. 3B we considered a threshold (*mean plus standard deviation* of responses in different combinations) and determined the border of the responses. Fig. 3C, 3D show tuning curves of same site with different adapters. It shows that adapter near the CF has stronger effect on the tuning curve of adapted sequence.

### Adaptation quantification

**Fig 4:**
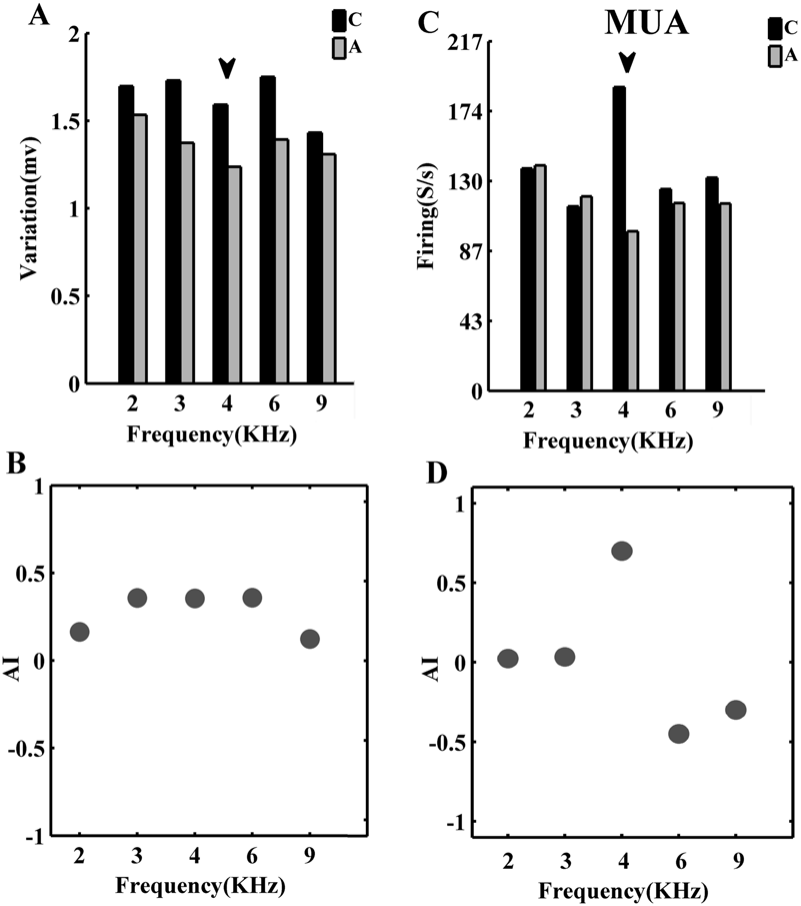
Quantification of the adaptation effect in a single site. A. The LFP variation in control sequence (black bar) and adapting sequences (gray bar) for a recording site. Flash mark indicates the adapter of the second sequence. B. Quantification of this variation by an index (AI). The positive value of AI shows that the neural response of a frequency in adapting sequence is weaker than that in control sequence. C. Comparison between firing rate of each frequency in control (black bar) and adapting (gray bar) sequences in MUA response for a recording site. This value was quantified by an adaptation index (AI) for all frequencies (D).

LFP variation and firing rate were used to enumerate the difference between responses of the two conditions. The Adaptation Index (AI) quantified the difference between responses of control and adapted sequences. It indicates the adaptation value and refers to the normal distance between responses of each stimulus in two control and adapted conditions. It was calculated for responses of each frequency at the level of 60 dB in two conditions (Fig. 4B, 4D). In each site, this criterion was calculated and averaged across all trials for the selected frequencies. AI values ranged between −1 and +1. The positive value of AI shows that the response of stimulus in adapted sequences is weaker than the response in control sequence. Therefore, it indicates the adaptation effect value. This measurement was calculated for both the LFP and MUA responses.

### Adaptation is Adapter-dependent

**Fig 5:**
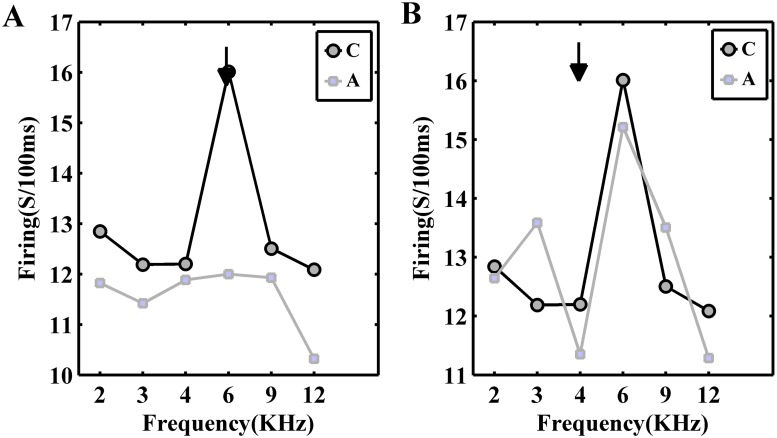
Adaptation effect is adapter dependence. A. The responses of adapter and the neighboring frequencies in a single site, in control (black line) and adapting (gray line) sequences (an isointensity curve at the adapter level) B. The same as (A) with a different adapter (frequency 4 kHz). Different adapter causes a different level of adaptation. In part (A) that the best frequency was selected as adapter, the reduction of adapting sequence in comparison to control sequence was more than this reduction in part (B). It shows that the responses of the second sequence in the same site with two different adapters, have different behaviour. The flash marks indicate the adapter location of the second sequence.

For finding the relation between adaptation and CF, we investigated the isointensity curve at the adapter level. In order to specify the adaptation effect on the neighboring frequencies, six frequencies were considered just at the adapter level (60 dB). For each frequency, the firing rate was calculated as the response strength in two conditions. Fig. 5, shows the isointensity curve of the selected frequencies at the same recording site with two different adapters. As it shows, the changes of responses of adapted sequence (adapting; gray line) is specially related to the adapter frequency. The adapter frequency, which was selected on CF, had stronger effect on adapting responses than when adapter is far from CF.

### Population results

In the previous section, the neural analyses were illustrated in a single site with two different adapters. In this section, the population results of 167 sites in A1 neurons are reported. The difference between these two conditions was evaluated by statistical measurement; Wilcoxon signed-rank test. In all parts, the difference between the two distributions has been compared with three ​​p-values (0.001, 0.01, and 0.05). Respectively, the significance of this difference has been indicated with one star, two stars, and three stars.

**Fig 6:**
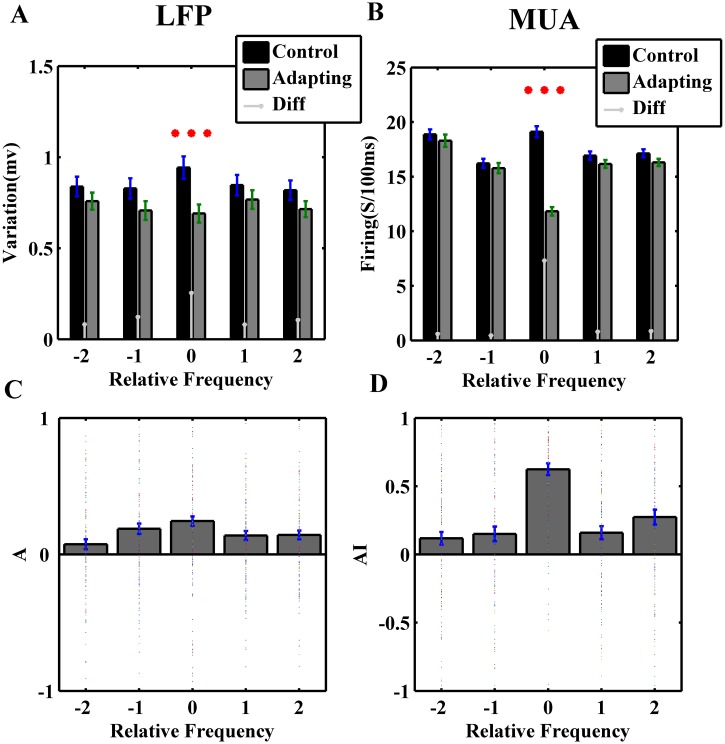
Adaptation decreases the strength of neural responses in A1 neurons. *A,B*. Comparison of the average of LFP voltage variations and spike count distributions of different frequencies f1–f5, when they were presented in the control sequence (black bars) or adapting sequence (gray bars) and the difference of them that was indicated by a narrow bar between them. Three stars indicate the significant difference between the two cases (P<0.001, Wilcoxon signed-rank test). *C*,D. Quantification criterion that shows the adaptation effect on the neural responses. Dot plots indicate the adaptation criteria value of neurons in different frequencies and the average of them has been shown by the bar plot (Error bar is the standard error). A is Adaptation Index that was achieved from the LFP analysis and AI is Adaptation Index that was calculated according to the firing rate. Positive valuesindicate that adaptation suppressed the neural responses. This suppression is significant in adapted frequency.

Considering the neural populations, the responses of adapted frequency (f3) and four neighboring frequencies (f1, f2, f4, f5) were aligned to each other in the two sequences. First, the variation of LFPs and value of spike counts for two conditions (control and adapting) were compared. This comparison indicated a significant difference in both LFP and MUA analysis, which was arisen in adapted response. In addition, a reduction in neighboring frequencies was seen but not conspicuous. This reduction is not significant but occurred in all neighboring frequencies. A narrow bar is employed in Fig. 6A and 6B to show the difference between each frequency in two sequences. The positive values of it in all frequencies indicate this reduction. Furthermore, in both LFP and MUA (Fig. 6C, 6D), positive value of adaptation index indicates this reduction in all frequencies. Each dot shows the AI value of each neuron that was averaged across its trials. In addition, the bars indicate the average value of them across neurons.

The significant suppression in adapter can be relevant to the special decrease founded in different auditory pathway from A1, IC, MGB, which is called SSA. Moreover, the response reduction in neighboring stimulus after the adaptation processes can be explained by the cross frequency adaptation concept that mentioned the effects of each stimulus on neighboring stimulus responses.

**Fig 7:**
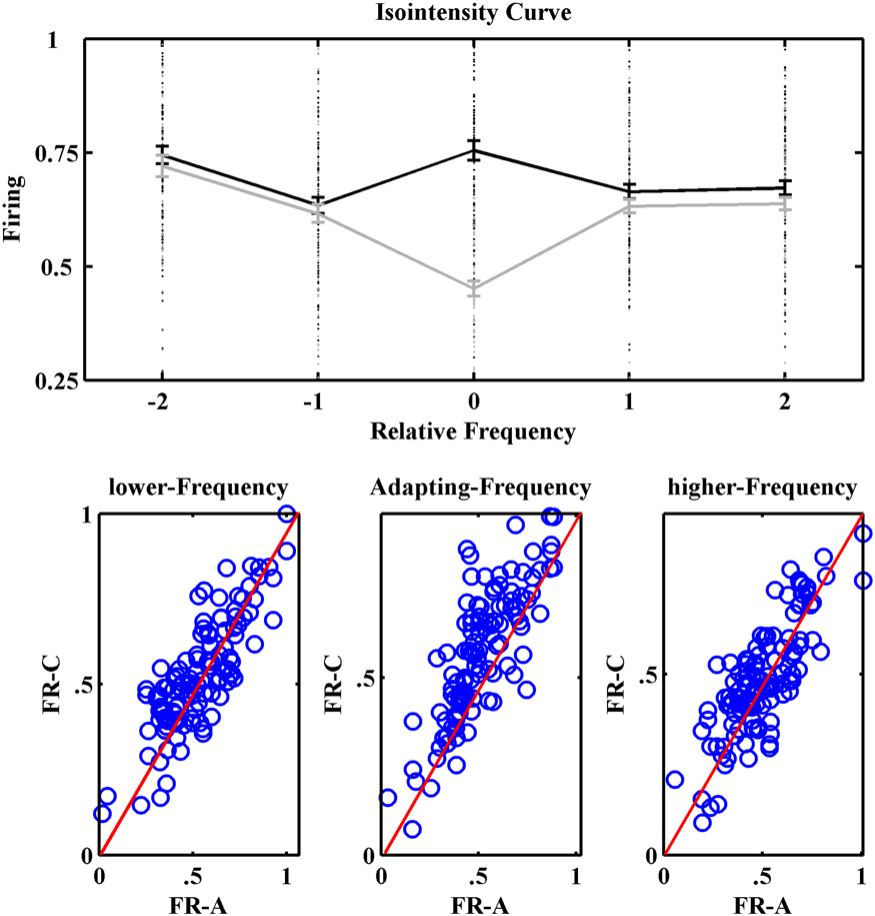
Isointensity curve and comparison between the responses of stimuli in the control and adaptation sequeces for the population of neurons. A. The population isointensity curve of 167 neurons that their firing rates were normalized and they were aligend to each other according to their adapter and four neighboring frequencies. Error bars are the standard error of the mean (s.e.m.). B. The scatter plots show the firing rate in control sequence vs. adapting sequence and the histogram of neural response changes for the adapted frequency (middle plot) and higher and lower neighboring frequencies in 60 dB. Dots indicate the neural responses of each neuron averaged across trials. Each dot placed upper the red line, demonstrates that the firing rate of that neuron in control sequence (FR-C) is more than the firing rate of it in adapting sequence (FR-A).

In addition, to show the effect of adaptation better, the isointensity curves of the two sequences in the neuronal population were used. In order to elucidate this effect, the spike counts of all neurons were normalized to the maximum firing rate. The responses of each neuron to the adapted and neighboring frequencies were characterized (dot plot in Fig. 7A). Then the average firing rate of each frequency was calculated across all neurons. Zero relative frequency demonstrates the adapted frequency. Moreover, the other integers show neighboring frequencies. In the isointensity curve of second sequence (gray line) a significant reduction in the adapted frequency was appeared in compared to the first sequence isointensity curve (black line). In Fig. 7B, the neuronal responses in two sequences were compared in the scatter plots for adapter and its neighboring frequencies. As it shows in the middle scatter plot, the power of adaptation shifts the responses of most neurons toward the higher firing rate in control sequence (FR-C) vs. firing rate of adapting sequence (FR-A).

### Adaptation shifts the tuning curves unevenly

In order to explore the adaptation effects on the tuning curves, the FRA and the tuning curve for two usual and adapted conditions (control and adapting sequences) were calculated. The FRA has been achieved with both LFP and MUA separately. For characteristic tuning curve in population, first all of the responses were normalized. Then LFP variations and spike counts considered as the strength of each stimulus.

**Fig 8:**
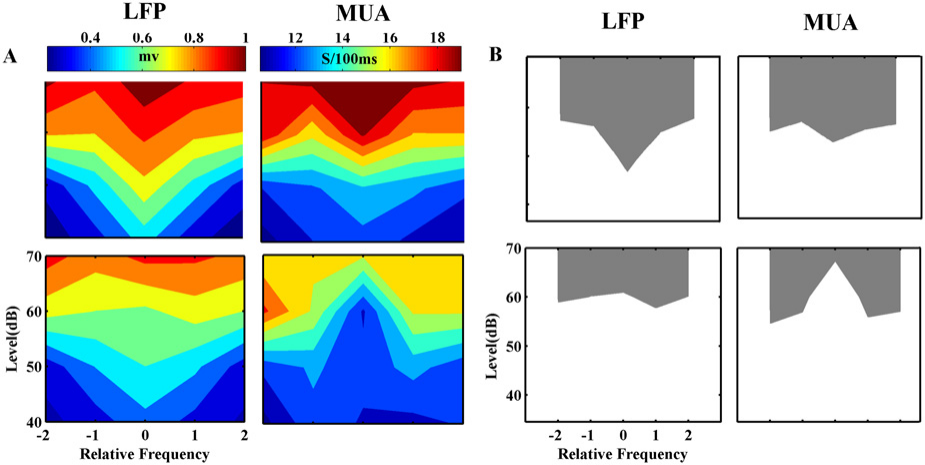
Adaptation effects on the FRA and the tuning curves. A. The frequency response area for average of 167 neurons in A1 area, which shows a decrease in their responses by adaptation. The responses of neurons in control sequence (up) and adapting sequence (down) that were calculated using LFP and MUA. B. The population tuning curves before (up) and after (down) adaptation. The relative frequency ‘0’ indicates the specific frequency that was used as adapter in the adaptation sequence.

The results indicate a suppression of neural responses of stimuli in adapting sequence and show a decrease in the response area. In the other words, in the adapting sequence, neurons respond to the frequencies in higher levels in compared to that frequencies in control condition sequence. This decrease is significant in the adapted frequency. Therefore, this adapter causes the tuning curves to shift toward the higher thresholds. The adaptation process changes the CF of the auditory tuning curve. Considering the neuronal population, it has elucidated that the tuning curve has been changed from one peak curve to multi peak curve. This concept is found conspicuously in the MUA tuning curves too (Fig. 8B).

### Adaptation strength and distance from adapter

**Fig 9:**
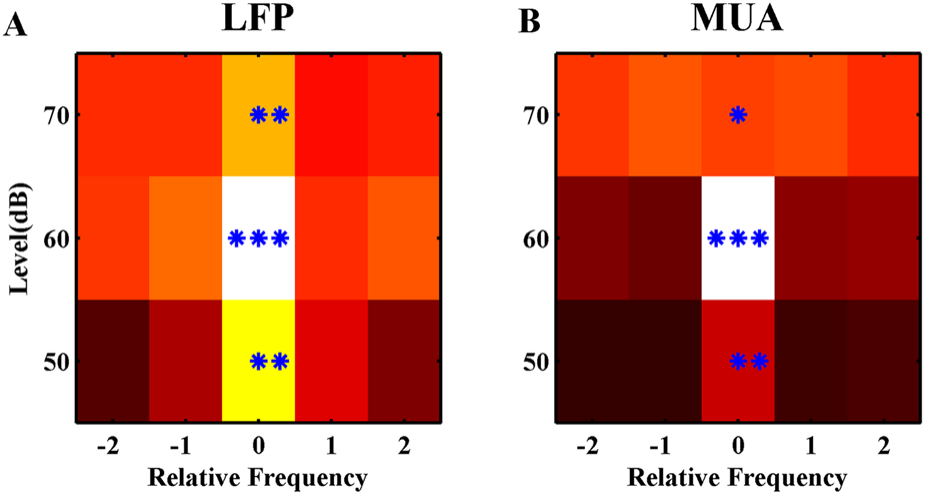
The difference between neural responses of adapted and control sequences. A. The distance btween control and adapted sequences responses in different combinations. The colors shows this distance from dark to white. Adapter response (stimulus with relative frequency ‘0’ and at the level of 60 dB) shows the largest decrease. Whatever, by distancing from adapter, this decreasing effect becomes lower. The adaptation effect is significant in the adapter and the combinations with the same frequency and different levels. (t-test; P<0.001_***, p<0.01_**, p<0.05_*).

The neural response of adapted sequence tuning curve was subtracted from the control one in order to quantify the FRA changes. This analysis has been performed in the case of adapted and four neighboring frequencies at three different intensities (Fig. 9). The contour curves show difference in the tuning curves of two sequences, which were calculated by both LFP and MUA. The results of LFP and MUA tuning curves indicate a significant difference in the case of adapted stimulus and the combinations of its frequency with one lower and one higher levels.

## Discussion

The effect of frequent presentation (adaptation) on a specific stimulus has been reported in different parts of auditory system [3,15,16,26]. In those studies, two-tone sequence was used to find out the effect of common or rare presentation of a specific stimulus on its neural response. We considered five-tone sequence for exploring the effects of repeated presentation of a special frequency on its and the peripheral stimuli responses. We investigated this effect on the tuning curves, which are achieved in the two sequences (control and adapted sequences).

The results suggest that adaptation to a specific frequency in a stream of audio events shifts the auditory tuning curves toward the higher-thresholds. This shift is uneven, since the adapter response was decreased more substantial than the neighboring stimuli. Therefore, the neural responses were suppressed in a sequence consisting of an adapter compared to their responses in a control sequence. This suppression changes the CF and the shape of the tuning curve.

### The effect of neighboring frequencies

According to a recent study [19], three controlling conditions were tested in addition to three usual blocks of oddball paradigms. The results discerned various in the neural responses of a special frequency in dissimilar sequences. This difference can occur because of the neighboring stimuli effects. In different parts of auditory pathway, decreasing the neural activities of a repeated sound stimulus has been investigated. However, what are the effects of adaptation to a special frequency in a stream of audio events, on the other stimuli in that sequence?

This study has been conducted to find out the effects of adaptation to a single stimulus (a pure tone with a special frequency and level) on the neighboring stimulus responses. Therefore, we measured both LFP and MUA of 167 recording sites in the primary auditory cortex of anesthetized rats. The voltage variation of LFP and the firing rate of MUA were compared for responses of each frequency in two states. In the first state, the responses of frequency-level combinations in a random presentation were considered for finding FRA. In the next state, a sequence was the same as the first one, just with this difference that a special frequency with 60dB level was considered as the adapted frequency. It was presented with the probability of 80% of all sequence stimuli. We find out that the adaptation to a specific frequency reduces the neural response of the combinations in that sequence. This reduction was significant for adapter frequency in the adapter intensity (60dB), in one-step higher, and in one-step lower level (Fig. 9). The results indicated that changing the frequency feature of a stimulus changes the sound more efficiently than changing the level variation.

### Comparison with other SSA paradigms

Several investigations have reported SSA in different parts of auditory pathway. Since a decade ago, the adaptation to a specific stimulus was indicated in the auditory cortex with an experiment of single unit recording. In addition, some similar behaviors have been extensively presented between MMN and adaptation to specific stimulus in a single neuron. This SSA increases the sensitivity of the neurons in front of unexpected changes. In next year’s, this phenomenon was exposed in other subcortical regions. It expresses a reduction in neural response of a repetitive stimulus in comparison with the rare presentation. Most of these studies considered the usual oddball paradigm that had been employed in MMN studies. In that paradigm, two probe stimuli (Standard and Deviant) were considered for investigation. In this paper, different combinations of pure tones have been considered in an adaptation paradigm to explore the effects of adaptation in the auditory tuning curves. The existence of different stimulus combinations affects the responses of these stimuli.

The population results show a reduction in neuronal responses in both LFP and MUA analyses. This suppression was not significant for the neighboring frequencies (t-test; p>0.05). This should probably be occurred because of the various dynamic behaviors of the neurons in different layers of neocortex [27]. Most of neurons decrease the responses of neighboring frequencies in a sequence with an adapted frequency. On the contrary, some neurons mostly tend to increase the neural responses of the neighboring frequencies [28]. Therefore, the non-significant suppression of neighboring stimuli, seen in the population results of this study, can be related to these oppositional behaviors. A recent study of adaptation in the visual system has been categorized the neurons in three types [28]. They found three different behaviors of the visual neurons in an adapted sequence. Accordingly, the categorization of neurons based on the adaptation strength can be used to investigate the adaptation changes in different neuron groups, which probably elucidates this effect much better.

On the other hand, several combinations of pure tones were needed to investigate tuning curves. Then, in order to have an adapter in seq.2, the probability of neighboring stimuli in seq.2 become less than their probability in seq.1. In fact, the tones in low probability presentation cause large responses [19]. Then because of the lower probability of neighboring stimuli in adapting sequence (seq.2), they were expected to have larger responses in comparison with control sequence (seq.1). This point weakens the suppression of the neighboring stimulus neural responses in seq.2 in comparison with seq.1.

The results show that adaptation to a specific frequency affects the neighboring frequencies; however, it depends on the adapter and its distance from the CF (Fig. 5). Furthermore, this effect has more strength in the same frequency of adapter with different levels compared to the same level of adapter with different frequencies (Fig. 9).

### Adaptation and inferior colliculus tuning curves

In this paper, we explored the effects of the adaptation to a specific frequency on the tuning curves of the auditory cortex. The auditory cortex is considered as a main part of auditory system in SSA phenomenon. According to the investigations, the tuning curves in A1 have several shapes [18]. This problem makes it difficult to find an effect on the neuronal population tuning curves. In addition, IC is one of the prominent area in the auditory pathway that has an essential role in SSA [25]. Subsequently, there is a near relation between auditory cortex and IC neurons. According to [15] the IC neurons tuning curves are in several shapes (near 70% of them are V shape). In [29] with a large population of IC neurons in anaesthetized guinea pig, it was shown that there are seven frequency response classes that were represented at all frequencies. So exploring the adaptation effects on the IC neurons tuning curves can be a controversial issue.

## Conclusions

In this study, the rats have been presented with two audio sequences as the stimulus while the neural activities have been recorded extracellularly from the area of primary auditory cortex. LFP and MUA results demonstrate that adaptation makes a shift on the tuning curve of A1 neurons toward the higher-level intensities. It elucidates that adaptation to one stimulus affects the neural responses of the neighboring stimulus. Generally, the population responses of all stimuli in the adapted sequence have been extensively suppressed. This reduction was significant in the response strength of adapter (P<0.001, Wilcoxon signed-rank test) which is likely relevant to the stimulus specific adaptation that is characterized by two-tones sequence in various studies. Moreover, it causes a reduction on the response of neighboring combinations (cross-frequency adaptation) but it is not significant. This effect depends on the adapter and its distance from CF.
